# *In Planta* Synthesis of Designer-Length Tobacco Mosaic Virus-Based Nano-Rods That Can Be Used to Fabricate Nano-Wires

**DOI:** 10.3389/fpls.2017.01335

**Published:** 2017-08-18

**Authors:** Keith Saunders, George P. Lomonossoff

**Affiliations:** Department of Biological Chemistry, John Innes Centre, Norwich Research Park Norwich, United Kingdom

**Keywords:** tobacco mosaic virus, transient expression, nano-rods, cobalt-platinum binding, nano-wires

## Abstract

We have utilized plant-based transient expression to produce tobacco mosaic virus (TMV)-based nano-rods of predetermined lengths. This is achieved by expressing RNAs containing the TMV origin of assembly sequence (OAS) and the sequence of the TMV coat protein either on the same RNA molecule or on two separate constructs. We show that the length of the resulting nano-rods is dependent upon the length of the RNA that possesses the OAS element. By expressing a version of the TMV coat protein that incorporates a metal-binding peptide at its C-terminus in the presence of RNA containing the OAS we have been able to produce nano-rods of predetermined length that are coated with cobalt-platinum. These nano-rods have the properties of defined-length nano-wires that make them ideal for many developing bionanotechnological processes.

## Introduction

Particles of Tobacco mosaic virus (TMV) consist of a 6.4 kb molecule of single-stranded RNA encapsidated by approximately 2130 copies of the 17.5 kDa coat protein arranged with helical symmetry. TMV particles are hollow cylinders 300 nm in length with external and internal diameters of 18 and 4 nm, respectively. TMV is probably the highest yielding plant virus and thus its particles have attracted considerable interest for use in bio- and nanotechnology. For example, peptides have been fused to exposed locations of the coat protein for multi-epitope display and particles have been both genetically and chemically modified. Given their rigid rod-shaped morphology, a particular potential use of TMV particles is the production of electrically conductive “nanowires” for the incorporation into nanoscale devices (for reviews of this technology see Lomonossoff and Evans, 2011; Fan et al., 2013).

Though large quantities of TMV rods can be produced by infecting plants with the virus, this approach suffers from a number of disadvantages, the principal of which, from the point of view of the production of nanowires, is that it is difficult to significantly modify the length of the particles without losing infectivity. In addition, there are limitations to the modifications that can be made to the viral coat protein before the productivity of the virus is compromised. As a result there have been several attempts to produce TMV-derived rods without the necessity for virus infection. These approaches have made use of the large amount of information that has been gleaned about the assembly process since the original demonstration that particles can be assembled *in vitro* through the mixing of the viral RNA and coat protein (Fraenkel-Conrat and Williams, 1955). It is known that assembly is initiated at a single “origin of assembly sequence” (OAS) which, in the case of the U1 or Vulgare strain, is positioned approximately 1 kb from the 3′ end of the genomic RNA (Zimmern and Wilson, 1976; Zimmern, 1977; Zimmern and Butler, 1977). Initiation of assembly requires the presence of a two-layer disk aggregate (Butler and Klug, 1971), containing 34 coat protein subunits, that interacts with a hairpin structure formed by the OAS. Assembly then proceeds bidirectional, (Butler et al., 1977) with assembly toward the 5′ end of the RNA being considerably faster than that toward the 3′ end (Lomonossoff and Butler, 1979, 1980; Butler, 1984).

Experiments using synthetic RNA molecules transcribed *in vitro* showed that it is possible to achieve encapsidation of essentially any RNA molecule by the TMV coat protein provided it contains the OAS (Sleat et al., 1986; Turner et al., 1989). It is possible to produce structures more complex than a linear rod by incorporating more than one copy of the OAS on a single RNA molecule resulting in multiple initiation events (Gallie et al., 1987; Eber et al., 2015). However, though highly successful in demonstrating the range of structures that can be assembled from RNA containing the TMV OAS and the TMV coat protein, the *in vitro* assembly approach has two disadvantages: it is costly to scale up and the coat protein has to be produced separately, usually from TMV-infected plants. This inevitably restricts the range of coat protein mutants that can be used to those which are compatible with the infection cycle. In an attempt to circumvent this limitation, Shire et al. (1990) expressed the TMV coat protein in *Escherichia coli*. The bacterially expressed protein was unable to assemble with TMV RNA *in vitro*, a problem which was ascribed to the lack of acetylation of the N-terminal serine preventing the formation of the disk structures necessary to initiate assembly. Hwang et al. (1994) co-expressed the TMV coat protein and a variety of RNA molecules containing the OAS, including full-length TMV genomic RNA, in *E. coli*. They showed that assembled rods could be detected and, when genomic TMV RNA was used, the purified material could infect tobacco plants. However, the assembly process appeared to be relatively inefficient, with some OAS-containing RNAs being only partially encapsidated. Hwang et al. (1994) hypothesized that this was due to 70S ribosomes binding to the 5′ end of the transcribed RNA, thereby interfering with assembly in the faster 3′–5′ direction. Using a similar approach, Kadri et al. (2013) expressed the TMV coat protein in the presence or absence of RNA molecules containing the OAS in both bacteria and yeast. They found that TMV-like rods were assembled but that the majority of the encapsidated RNA was of host origin and the length distribution of the rods was similar whether or not OAS-containing RNA was present. Thus this approach was not an efficient way of producing rods of predetermined lengths and the authors speculated on why this was the case. One possible reason for the relatively inefficient encapsidation of the OAS-containing RNA is that the OAS was positioned such that the majority of assembly would have to occur in the relatively slow 5′–3′ direction.

Given the relative lack of success obtained previously using heterologous *in vivo* assembly systems, we decided to examine the possibility of producing defined length rods by transient expression in plants. Since assembly of TMV-like particles has previously been reported in transgenic tobacco plants (Sleat et al., 1986, 1988; Gallie et al., 1987), we rationalized that a plant-based system may be an efficient way of producing rods of predetermined length (Sainsbury and Lomonossoff, 2008). Using the pEAQ-*HT* vector system (Sainsbury et al., 2009) to express both the TMV coat protein and RNA molecules with the OAS positioned near the 3′ terminus, we demonstrate the efficient production of rods of predetermined length and, furthermore, by the expression of variant coat protein, the generation of nano-rods that promote the binding of cobalt-platinum to the exterior surface, thereby producing conducting nano-wires.

## Materials and Methods

### Molecular Cloning

#### pEAQ-*HT*-TMV-CP/OAS, pEAQ-*HT*-TMV-CP(CP9)/OAS, and pEAQ-*HT*-TMV-CPHis/OAS

**FIGURE 1:**
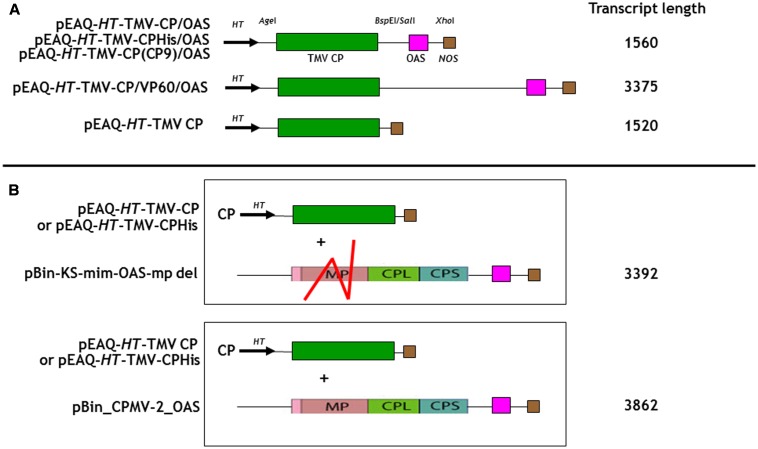
Schematic representation of the infiltrated coat protein gene and OAS element containing gene constructions for **(A)** *In cis* and **(B)** *in trans* infiltrations. The predicted transcript length for each construct is listed calculated from the start of transcription at the 35S promoter to its termination by the *nos* terminator, Meshcheriakova et al. (2014). TMV CP, green box; the OAS element, pink box and the *nos* terminator, brown box are indicated. The location of the *HT*, hyper translatable CPMV 5′ UTR sequence (arrowed) is indicated (Sainsbury and Lomonossoff, 2008). Red lines indicate the approximate location of the deleted region of the CPMV movement gene (MP) in pBin-KS-OAS-mp del. CPL and CPS, sequences encoding the CPMV large and small capsid proteins. The locations of relevant restriction sites in the gene constructs are shown.

A DNA molecule comprising of the TMV *Vulgare* strain coat protein sequence (nucleotides 5595–6069; Goelet et al., 1982; GenBank accession number V01408.1), linked to the OAS sequence (nucleotides 5393 – 5578; Goelet et al., 1982) via a linker containing *Bsp*EI and *Sal*I restriction sites and containing flanking *Age*I and *Xho*I sites was synthesized (GeneArt), (**Supplementary Figure S1A**). After digestion with *Age*I and *Xho*I this was ligated into similarly digested pEAQ-*HT* (Sainsbury et al., 2009) to yield pEAQ-*HT*-TMV-CP/OAS (**Figure 1A**). pEAQ-*HT*-TMV-CP(CP9)/OAS was constructed in a similar manner (Gene Art), (**Supplementary Figure S1B**) but with the addition of a sequence encoding the peptide CNAGDHANC^∗^ (Mao et al., 2004) after the carboxyl terminal amino acid of the TMV coat protein sequence (**Figure 1A**). Two oligonucleotides, KS 109 and KS 108 (**Table 1**) were synthesized to clone via PCR with Phusion DNA polymerase (New England Biolabs), a TMV coat protein possessing C-terminal six histidine residues. pEAQ-*HT*-TMV-CP/OAS was used as a DNA template for this PCR reaction. The wild-type coat protein sequence in pEAQ-*HT*-TMV-CP/OAS was then replaced with the modified coat protein sequence via its *Age*I and *Sal*I restriction sites (**Figure 1A**) to yield pEAQ-*HT*-TMV-CPHis/OAS.

**Table 1:** The Sequence of the cloning oligonucleotides.

Primer	Sequence
KS 109	5′-AATTCGCGACCGGTTTTAAATATGTCTTACAGTATCACTACTCC
KS 108	5′-TATATATACTCGAGCTATCAGTGATGGTGATGGTGATGAGTTGCAGGACCAGAGGTCC
KS 91	5′-TTATAGCCGGCTAATATGGAGCAAAACTTGTTTGGCC
KS 92	5′-TTAACGTCGACCTTTCATTAAGCAGCAGTAGCAGTGTGTC
KS 111	5′-GTTTGAGAGAAGATTACAAACG
KS 112	5′-TAATACGACTCACTATAGGGCGGTTCGAGATCGAAAC


#### pEAQ-*HT*-TMV-CP and pEAQ-*HT*-TMV-CPHis

The OAS of pEAQ-*HT*-TMV-CP/OAS and pEAQ-*HT*-TMV-CPHis/OAS were removed by digestion with *Sal*I and *Xho*I (**Supplementary Figure S1A**). The resulting DNA was self-ligated to create pEAQ-*HT*-TMV-CP and pEAQ-*HT*-TMV-CPHis.

#### pEAQ-*HT*-TMV-CP/VP60/OAS, pBin-KS-mim-OAS-mp del, and pBin_CPMV-2_OAS

The cowpea mosaic virus (CPMV) VP60 coding region DNA was derived from pBinPS2NT (Liu and Lomonossoff, 2002) by PCR amplification with Phusion DNA polymerase (New England Biolabs) using primers KS 91, and KS 92 (**Table 1**). The resulting DNA was cloned via the *Ngo*MIV and *Sal*I restriction sites, (underlined) into *Bsp*EI and *Sal*I digested pEAQ-*HT*-TMV-CP/OAS to yield pEAQ-*HT*-TMV-CP/VP60/OAS. DNA corresponding to TMV nucleotides 5420–5546 inclusive (encompassing the OAS, Goelet et al., 1982) was synthesized (GeneArt) and cloned into the movement-deficient CPMV RNA-2 vector pBIN-mim-AvrII-mp del (Madi et al., 2015) via its *Apa*I and *Avr*II restriction sites to yield pBin-KS-mim-OAS-mp del. *Bam*HI and *Not*I, unique restriction sites both present in pBin-KS-mim-OAS-mp del and pBin_Mimic_AIV, a derivative of pBinP-EMS-11 (King et al., 2007) were utilized to restore the movement-deficient CPMV property in pBin-KS-mim-OAS-mp del to yield pBin_CPMV-2_OAS.

The DNA sequence of all the modified gene constructs were confirmed by DNA sequencing (Eurofins genomics). Verified pEAQ-*HT* clones were used to transform *Agrobacterium tumefaciens* LBA4404 and were subsequently used for plant infiltration experiments (Sainsbury et al., 2014). Infiltration experiments were performed with *Agrobacterial* cultures at a density of 0.4 OD units/mL in MMA buffer (Sainsbury et al., 2014). Equal volumes of cultures at this density were mix in order to perform co-infiltration experiments.

### Isolation of Nano-Rods from Infiltrated Plant Leaves

*Agrobacterium tumefaciens* infiltrated leaf tissue was harvested 8 days post infiltration and homogenized in three volumes of extraction buffer (100 mM sodium phosphate, pH 7.0). After squeezing through Miracloth (Calbiochem), the extracts were clarified by centrifugation at 13,000 × *g* for 20 min at 4°C. To improve the quality of the resulting nano-rods, the supernatant was incubated with one quarter volume of CHCl_3_ and mixed at this stage and an additional centrifugation step of 13,000 × *g* for 20 min at 4°C was performed. To the resulting supernatant was added one quarter volume of a 20% (w/v) PEG 6000, 1 M NaCl solution and the mixture was stirred overnight in a cold room. Protein was sedimented at 13,000 × *g* for 20 min at 4°C and the pellet was re-suspended in 10 mM sodium phosphate pH 7.0 and further clarified at 27,000 × *g* for 20 min at 4°C. Nano-rods in the resulting supernatant were recovered by centrifugation at 118,700 × *g* for 3 h at 4°C, re-suspended in 10 mM sodium phosphate pH 7.0 and were subjected to centrifugation for 24 h at 209,627 × *g* (10°C) in a CsCl gradient formed of equal volumes of 42, 49, 57, and 65% (w/v) CsCl in 10 mM sodium phosphate pH 7.0. After centrifugation, the gradients were illuminated with white light from directly above and photographed. Nano-rod-containing bands were removed from the CsCl gradient by side puncture with a syringe and the material was dialysed against H_2_O overnight in a cold room.

### Resolution of Coat Protein by Gel Electrophoresis

Protein samples were denatured in NuPAGE sample buffer (Invitrogen) supplemented with 5% (v/v) 2-mercaptoethanol and by subsequent heating to 95°C for 5 min. Denatured proteins were resolved on 12% (w/v) NuPAGE polyacrylamide gels run with MOPS buffer (Invitrogen). See Blue Plus, protein molecular weight markers (Invitrogen) were run on all NuPAGE gels. Instant Blue stain (Expedeon Ltd.) was used to visualize the protein bands. Western blotting to detect either TMV coat protein with an anti-TMV antibody, (AS-72203-2ML, Austral Biologicals, San Ramon, CA, United States) or the six C-terminal histidine residues with a monoclonal antibody prepared to six histidine residues, (Cat 34660, Qiagen) was performed as described by Saunders and Lomonossoff (2015).

### Analysis of RNA Encapsidated within Nano-Rods

Encapsidated RNA was isolated from purified nano-rods by extraction with phenol/CHCl_3_ with the addition of 0.2 volume 10% (w/v) SDS and 0.1 volume 3 M Na acetate pH 4.8. After mixing and centrifugation at 16000 × *g* for 10 min, the RNA in the aqueous phase was recovered following overnight precipitation at -20°C with the addition of 2.5 volume of ethanol and by centrifugation at 16,000 × *g* for 20 min. RNA pellets were washed with 70% (v/v) ethanol and after centrifugation at 16,000 × *g* for 20 min, the RNA was air-dried and suspended in 20 μL H_2_O. 5 μL of RNA was denatured by the addition of formaldehyde (0.7 vol) and formamide (2 volume), incubated at 65°C for 5 min and subsequently resolved in 1.2% (w/v) agarose gel containing 2.2 M formaldehyde. The RNA size marker, catalog number 15623-200 (Invitrogen) was run alongside the extracted RNA samples and the RNA detected under UV light. For northern blot analysis, the RNA resolved in an agarose gel was stained by ethidium bromide and subsequently transferred to a positively charged nylon membrane by overnight capillary transfer in 20 × SSC (3 M NaCl, 300 mM Na citrate). The RNAs containing the OAS sequence were detected by hybridisation with a complementary 126 nucleotide DIG-labeled RNA probe (Meltzer et al., 1998) containing the OAS sequence, (nucleotides 5546 to 5420, Goelet et al., 1982). This was prepared by T7 RNA transcription of a PCR product made using pEAQ-*HT*-TMV CP/OAS as the template DNA with the primers KS 111 and KS 112, (**Table 1**), the latter possessing the T7 RNA polymerase promoter sequence.

### Transmission Electron Microscopy

Particle preparations were spotted onto carbon-coated copper grids and when necessary were negatively stained with 2% (w/v) uranyl acetate. The grids were examined using a Tecnai 20 transmission electron microscope. Nano-rod length of TMV rod-like structures that clearly resembled TMV-like particles, was determined by utilizing AMT camera software version 3.2 (Supplementary Table S1).

### Cobalt Platinum Binding

After gradient purification, a volume of nano-rods were incubated at room temperature with an equal volume of a 1:1:1: solution of 50 mM CoCl_2_, 50 mM H_2_PtCl_6_ and 100 mM NaBH_4_ for 22 or 69 h with constant agitation. Nano-rods were spun-washed at 3,000 × *g* with 1 mL H_2_O in Microcon YM-100 MWCO 100 kDa spin filters (Millipore) prior to electron microscopy.

## Results and Discussion

### Creation of an “*In cis*” Assembly System

As a first step to creating defined length nano-rods, we designed a DNA sequence that included the TMV coat protein open reading frame with a TMV OAS element to its 3′ side. This reverses the order of the OAS and coat protein (CP) that is found in TMV U1 genome and was done to ensure that the majority of RNA encapsidation would occur in the fast 3′–5′ direction. Upon transcription, this gene construct would direct the synthesis of TMV CP which will subsequently interact with the OAS element to form nano-rods. As both the CP and OAS are present on the same RNA, we term this the *in cis* approach. This synthetic DNA was cloned into pEAQ-*HT* (**Figure 1A**), a plant-based expression vector that produces abundant quantities of highly translatable RNA (Sainsbury et al., 2009) to yield pEAQ-*HT*-TMV CP/OAS. The predicted length of the RNA transcribed from this construct is 1560 nucleotides, including sequences derived from the pEAQ vector (Meshcheriakova et al., 2014), approximately one quarter of the length of genomic TMV RNA. Subsequently an additional stretch of nucleic acid from the unrelated plant virus, cowpea mosaic virus, was placed between the TMV coat protein and OAS elements, to give construct pEAQ-*HT*-TMV-CP/VP60/OAS which is predicted to give a transcript of 3375 nucleotides, approximately twice as long as that from pEAQ-*HT*-TMV CP/OAS. As a control a further construct, pEAQ-*HT*-TMV CP, was created which was designed to produce RNA encoding the TMV CP but which lacks the OAS. (**Figure 1A**).

**FIGURE 2:**
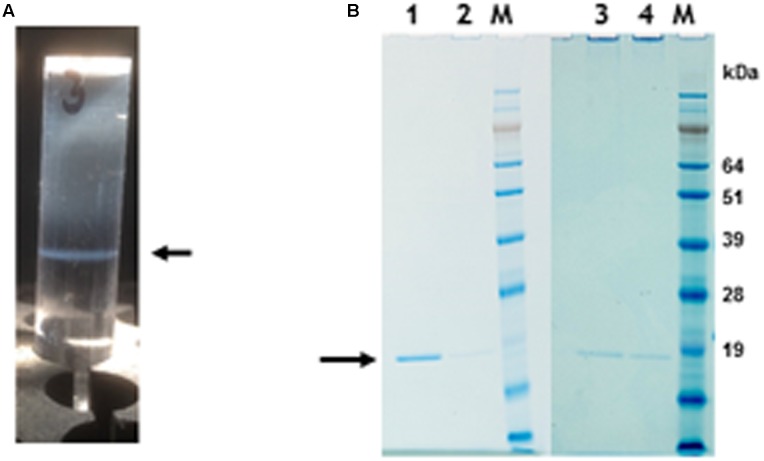
Protein gel analysis and CsCl gradient separation of nano-rods. **(A)** CsCl gradient resolution of pEAQ-*HT*-TMV CP/OAS nano-rods. Arrow indicates the position of the nano-rods in CsCl gradient. **(B)** NuPAGE gel analysis of the coat protein after PEG purification of nano-rods. Lane 1, pEAQ-*HT*-TMV CP/OAS. Lane 2, pEAQ-*HT*-TMV CP/VP60/OAS. Lane 3, co-infiltration of pEAQ-*HT*-TMV/CP and pBin-KS-mim-OAS-mp del. Lane 4 co-infiltration of pEAQ-*HT*-TMV/CP and pBin_CPMV-2_OAS. M, protein marker standards. Arrow indicates the position of TMV CP. Original images are shown in **Supplementary Figure S4**.

To determine if nano-rods could be formed from the above constructs, *Nicotiana benthamiana* leaves were separately infiltrated and extracts were subjected to the standard TMV purification protocol including precipitation with PEG and ultracentrifugation, followed by isopycnic centrifugation on caesium chloride (CsCl) gradients. Material banding at the same density as an authentic TMV control was collected and the CsCl removed by dialysis. **Figure 2A** shows the typical position in a CsCl gradient of the nano-rods resulting from the infiltration of plant leaves with pEAQ-*HT*-TMV CP/OAS. NuPAGE gel electrophoresis and Instant Blue staining revealed the presence of a single band of the size expected for TMV coat protein in the nano-rod-containing fractions of the CsCl gradients in the case of pEAQ-*HT*-TMV-CP/OAS and pEAQ-*HT*-TMV-CP/VP60/OAS (**Figure 2B**, lanes 1 and 2). The identity of this protein was confirmed by MALDI-TOF analysis of tryptic digests of the material in the bands (data not shown). By contrast, no band corresponding to TMV CP could be seen in extracts prepared from leaves infiltrated with pEAQ-*HT*-TMV CP, a construct that is designed to produce TMV CP but no OAS-containing transcripts. These results indicate that expression of both the TMV CP and the presence of an RNA containing the OAS is essential for the formation of TMV nano-rods and that the buoyant density of such artificial rods in CsCl is similar to that of native TMV. This indicates that the nano-rods have a similar RNA:protein ratio, and therefore structure, to native TMV. The main difference between the results obtained with pEAQ-*HT*-TMV-CP/OAS and pEAQ-*HT*-TMV-CP/VP60/OAS was the level of CP detected in latter which was only about 10% of that for the shorter construct in samples from which equal masses of leaf material was processed.

**FIGURE 3:**
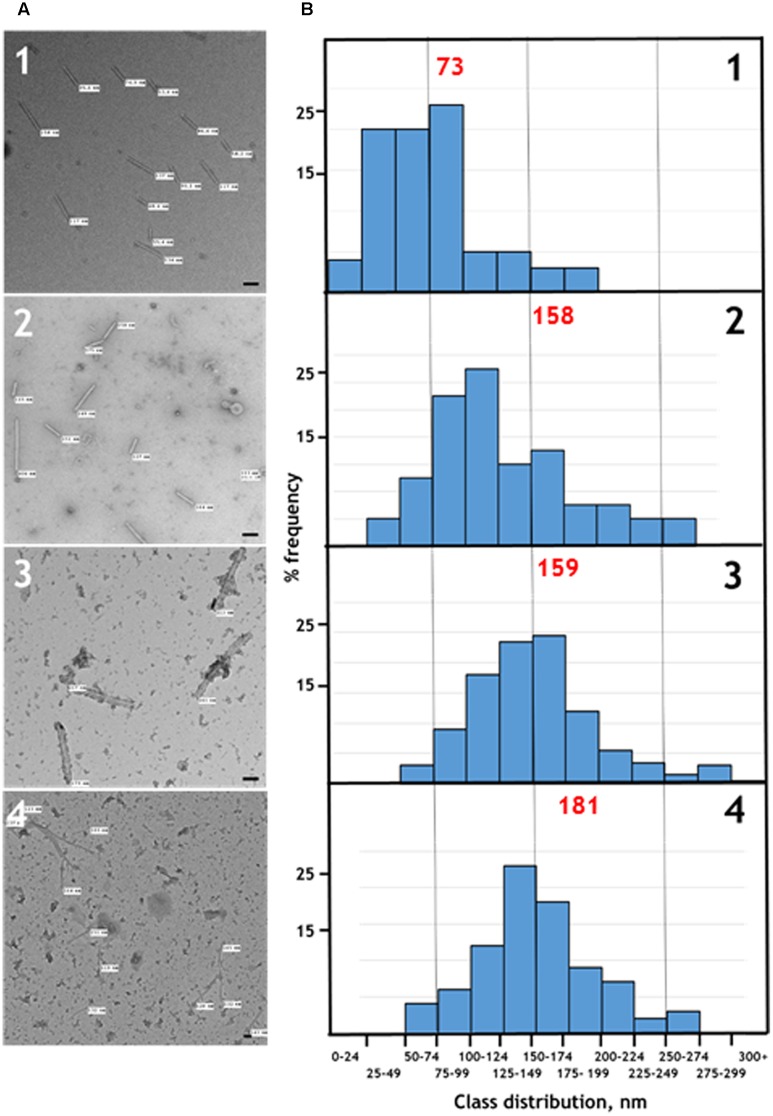
**(A)** Transmission electron microscopy, and **(B)** distribution of the measured lengths of the generated nano-rods. Nano-rods generated from infiltrations with, 1 – pEAQ-*HT*-TMV CP/OAS, 2 – pEAQ-*HT*-TMV CP/VP60/OAS, 3 – pEAQ-*HT*-TMV CP and pBin-KS-mim-OAS-mp del, 4 – pEAQ-*HT* TMV CP and pBin_CPMV-2_OAS. The length of measured nano-rods, resulting from each infiltration is indicated in **(A)**. Scale bar = 50 nm. Red numbers in **(B)** indicate the theoretical length of the nano-rods arising from each infiltration.

To confirm that the TMV CP was incorporated into TMV-like rods of defined length the particles were examined by transmission electron microscopy using negative staining. The particles appeared to have identical external and internal diameters to native TMV particles but in both cases were shorter (**Figure 3A**, panels 1 and 2). The length of a TMV particle is dependent upon the length of its encapsidated genome with one TMV coat protein subunit interacting with three nucleotides. Therefore, the expected length of each type of nano-rod can be calculated from the number of nucleotides in the encapsidated RNA. The length of a representative number of particles was measured from the micrographs in **Figure 3A** and there was very good agreement between the calculated and measured lengths for each class of nano-rod (**Figure 3B**, panels 1 and 2), confirming our hypothesis that it should be possible to vary the size of nano-rods by changing the length of the RNA containing the OAS.

### Creation of an “*In trans*” Assembly System

Although the “*in cis*” approach described above was capable of producing nano rods of predetermined size it lacks flexibility. The production of the TMV CP from the same RNA that contains the OAS means that there is a minimum length of particle that can be produced and it is possible that encapsidation of the mRNA for the CP may adversely affect its translation. This, and increasing the length of the RNA to be encapsidated, may lead to insufficient CP being produced to completely encapsidate the OAS-containing RNA. We suspect that either or both of these phenomena are the cause of the low yield of nano-rods from pEAQ-*HT*-TMV-CP/VP60/OAS compared with pEAQ-*HT*-TMV-CP/OAS. Furthermore, it would be difficult to produce defined length nano-rods containing several CP variants if both the OAS and the CP sequence are on the same RNA.

To investigate the possibility of *in trans* assembly in which the CP and the OAS are on separate RNA molecules, the OAS element was introduced into a deleted (Madi et al., 2015) and a full-length version of CPMV RNA-2 (Liu and Lomonossoff, 2002) to give constructs pBin-KS-mim-OAS-mp del and pBin_CPMV-2_OAS, predicted to produce transcripts of 3392 and 3862 nucleotides, respectively, *in planta*, (**Figure 1B**). To examine the ability of these RNAs to assemble into rods, they were independently co-infiltrated with the pEAQ-*HT*-TMV CP gene construct (lacking the OAS) to create an *in trans* viral assembly system, (**Figure 1B**). Isolation and examination of the rods formed was carried out as described for the *in cis* system (**Figure 2B**, lanes 3 and 4). Negatively stained images of the particles indicated that the *in trans* system gave a mean rod length close to the expected length in the case of pBin-KS-mim-OAS-mp del (152 nm vs. a calculated length of 159 nm) whereas the rods produced from pBin_CPMV-2_OAS where somewhat shorter than expected (149 nm vs. 181 nm; **Figure 3**, panels 3 and 4). We do not know the reason for this discrepancy but it may reflect the lack of sufficient CP to encapsidate the longer RNA. However, it may also be due to the fact that two gene constructs are needed for nano-rod formation with the *in trans* system in contrast to *in cis* nano-rod formation where only a single gene construct has to be present in a transformed cell. As shown previously, co-infiltration is a less efficient process than infiltration with a single construct (Montague et al., 2011). The variation of the measured length of the nano-rods produced either from single or co-infiltrations (*in cis* or *in trans* nano-rod formation, respectively) in our study (**Figure 3B**), is in keeping with the variation measured and reported by Shukla et al. (2015) concerning the *in vitro* formation of nano-rods. In this previous study, *in vitro* synthesized RNA of a known length was incubated with purified TMV CP for up to 48 h to form nano-rods. Similarities in the variation of the lengths of the nano-rods are apparent for both systems. Indeed the *in vitro* study reports that there is a greater variation in the length of the nano-rods resulting from the incubation of the longest RNAs when presented to CP, unlike the current investigation where a similar degree of variation in the length of the nano-rod is observed irrespective of its predicted length.

### Confirmation That Transcript Length Determines Particle Length

**FIGURE 4:**
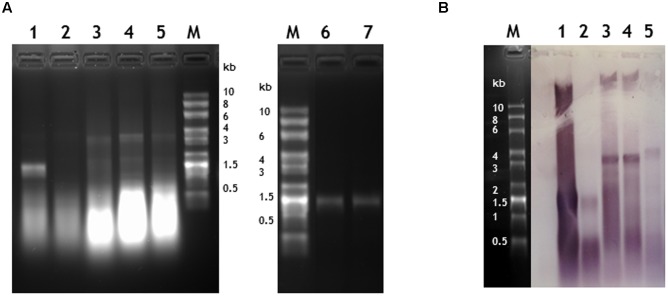
Verification of the length and sequence of RNA molecules encapsidated in nano-rods. Denaturing agarose gels of RNA extracted from nano rods either stained with ethidium bromide **(A)** or blotted and probed in a northern blot with an anti-sense OAS RNA probe **(B)**. RNA samples were extracted from the resulting nano-rods of plant infiltrations of 1- pEAQ-*HT*-TMV CP/OAS, 2- pEAQ-*HT*-TMV CPHis/OAS, 3- pEAQ-*HT*-TMV CP/VP60/OAS, 4- pEAQ-*HT* TMV CP + pBin-KS-mim-OAS-mp del. 5- pEAQ-*HT* TMV CP + pBin_CPMV-2_OAS. These samples were prepared without the CHCl_3_ step in the isolation procedure and the final CsCl gradient. Approximately 400 ng of RNA was run in each lane for each infiltration. 6- 330 ng and 7- 1 μg of RNA extracted from nano-rods of plants infiltrated with pEAQ-*HT*-TMV CP/OAS and isolated with a CHCl_3_ step and the final CsCl step in the procedure. The C terminus of the coat protein in lane 2 contained an additional six histidine residues. The original photograph of **(A)** is shown in **Supplementary Figure S5** and similarly of **(B)** in **Supplementary Figure S6**.

When RNA was extracted from particles purified without the CsCl gradient step, bands corresponding to RNA of the expected length, ranging in size between 1.5 and 3.8 kb could be seen on denaturing agarose gels stained with ethidium bromide apart from the sample produced from plants infiltrated with pEAQ-*HT*-TMV-CPHis/OAS (**Figure 4**, lanes 1 to 5). This construct was designed to express TMV CP modified to contain six histidine residues at its C-terminus (see below); this observation suggests that this tagged CP is not as assembly competent as wild-type (WT) CP. In all cases the samples of RNA extracted from partially purified nano-rods also contained substantial quantities of low molecular weight nucleic acid migrating more quickly than the 0.5 kb marker. To determine if this material was encapsidated into nano-rods or represented co-purifying host or *Agrobacterium*-derived nucleic acid, RNA was extracted from a sample of nano-rods obtained by infiltration pEAQ-*HT*-TMV-CP/OAS that had been purified from a CsCl gradient (**Figure 4A**, lanes, 6 and 7). In this case, a single band migrating close to the 1.5 kb marker could be seen – a size consistent with the encapsidation of the OAS-containing transcript from pEAQ-*HT*-TMV-CP/OAS. Significantly none of the shorter nucleic acids banded at the density of nano-rods suggesting that they are not encapsidated but rather represent nucleic acid fragments that are co-precipitated during the PEG precipitation step.

To confirm that the high molecular weight bands seen in the ethidium bromide-stained gel (**Figure 4A**, lanes 1–5) represented encapsidated RNA containing the TMV OAS, a replica agarose gel was subjected to northern blot analysis using a probe specific for the OAS. The specificity of the probe was confirmed by its ability to readily detect TMV RNA but not commercially available RNA size markers (**Supplementary Figure S6C**). In all cases, the high molecular weight RNA extracted from nano-rods hybridized with the OAS-specific probe to a degree consistent with the intensity of the ethidium bromide-stained bands; in contrast the lower molecular weight material hybridized less well, thereby suggesting that much of this material was not TMV-specific. In addition a high molecular weight band of hybridizing material could be seen in all samples (**Figure 4B**) which we believe represents plasmid DNA which has co-precipitated with the nano-rods. One significant difference between the results obtained by northern blotting and ethidium bromide staining was that there was evidence from the former that the RNA transcribed from pEAQ-*HT*-TMV-CPHis/OAS could be incorporated into nano-rods by the coat protein containing a 6 × His sequence at its C-terminus albeit less efficiently that the equivalent WT construct. The results of the study of the RNA encapsidated within the nano-rods suggest rods are likely to be of a more precisely defined size than implied by the TEM analysis of the negatively stained particles. This difference is probably a reflection of the harsh nature of the TEM analysis leading to shearing of some of the particles on the grids.

### Incorporation of Modified Coat Proteins into Nano-Rods

In addition to controlling particle length, another potential advantage of using transient expression to produce nano-rods is the possibility of expressing CP variants which may be incompatible with a productive infection. To examine this possibility, two CP variants with additional sequences at the C-terminus were produced, one with the addition of six histidines and the second with the sequence CNAGDHANC. The incorporation of six histidines has been shown to permit the assembly of the modified CP into a number of protein-only structures but the modified protein has been reported to be poorly incorporated into RNA-containing rods (Bruckman et al., 2011; Wnek et al., 2013). The CNAGDHANC peptide was originally identified as being able to bind cobalt- platinum (CoPt) when expressed on the surface of the coat protein of M13 bacteriophage (Mao et al., 2004) and has subsequently been used to promote the deposition of CoPt onto the surface of the spherical plant virus, cowpea mosaic virus (Aljabali et al., 2011). In both cases the sequence of the C-terminally modified CP was substituted for the wild-type CP in construct pEAQ-*HT*-TMV-CP/OAS to give pEAQ-*HT*-TMV-CPHis/OAS and pEAQ-*HT*-TMV-CP(CP9)/OAS (**Figure 1A**). Both constructs were designed to encapsidate an RNA of 1.5 kb producing nano-rods of around 75 nm. pEAQ-*HT*-TMV-CPHis, similar in design to pEAQ-*HT*-TMV-CP but possessing six C-terminal histidines, was also constructed to investigate *in trans* incorporation (**Figure 1B**).

**FIGURE 5:**
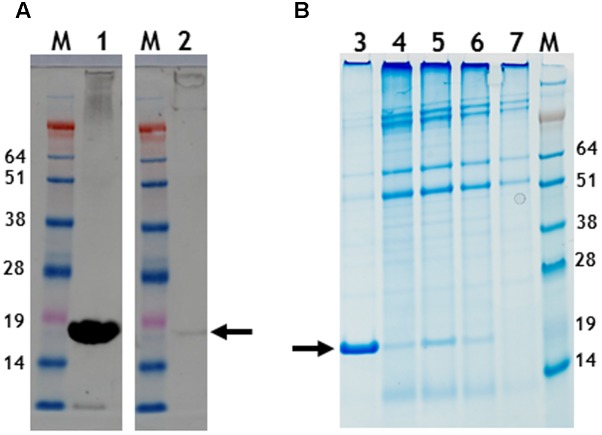
Gel migration and western blot detection of 6 histidine C-terminal possessing nano-rods. **(A)** Detection of CP with a polyclonal anti-TMV antibody., Lane 1, pEAQ-*HT*-TMV CP/OAS and lane 2, pEAQ-*HT*-TMV CP/His/OAS. Equal masses of leaf material was processed for each gene infiltration. **(B)** Instant blue staining of PEG purified nano-rods. pEAQ-*HT-*TMVCP/OAS, lane 3. pEAQ-*HT*-TMV-CPHis/OAS, lane 4. Co-infiltration of pBin-KS-mim-OAS-mp del and pEAQ-*HT*-TMV-CP lane 5. Co-infiltration of pBin-KS-mim-OAS-mp del and pEAQ-*HT*-TMV-CPHis lane 6. Co-infiltration of pBin-KS-mim-OAS-mp del, pEAQ-*HT*-TMV/CP and pEAQ-*HT*-TMV/CPHis, lane 7. Arrows indicate the position of resolved CP. The original photographs for (A,B) are shown in **Supplementary Figure S7**.

In the case of pEAQ-*HT*-TMV-CPHis/OAS, the yield of nano-rods obtained after PEG precipitation of extracts from leaves infiltrated with pEAQ-*HT*-TMV-CPHis/OAS was about 1% of that obtained from leaves infiltrated with pEAQ-*HT*-TMV-CP/OAS, judged by the detection of CP (arrowed) by western blotting with a polyclonal antibody to TMV (**Figure 5A**). Here equal masses of infiltrated leaf material harvested at 7 days post infiltration from both infiltrations was processed. Instant Blue-stained gels of preparations of nano-rods isolated by PEG precipitation (**Figure 5B**), showed the presence of CP suggesting that the modified CP could be assembled into nano-rods via the *in trans* reaction, (**Figure 5B**, lane 6) This substantiated the finding above, concerning the detection of encapsidated RNA in the *in cis* derived six histidine rods by northern blotting, (**Figure 4A**). This was confirmed by TEM analysis which showed the presence only a few nano-rods of the expected size (data not shown). An attempt to incorporate the six histidine C-terminal CP into nano-rod structures via the *in trans* reaction upon the co-infiltration of both the WT and the six histidine CP coat protein constructs were fruitless, (**Figure 5B**, lane 7). This again highlights the difficulty of incorporating this modified CP into RNA containing rods, discussed above. However, the Instant Blue stained gel, (**Figure 5B**, lane 6), revealed the presence of CP in PEG purified nano-rods (generated via the *in trans* reaction). Thus, it is likely that the C-terminal six histidines have been removed probably by protease digestion thereby allowing for the formation of WT nano-rods. Taken together, these results show that assembly of the His-tagged protein into RNA-containing nano-rods is inefficient compared to the incorporation of wild-type coat protein. This may be because of the previously noted propensity of the His-tagged coat protein to assemble into protein-only aggregates rather than nucleoprotein rods (Bruckman et al., 2011; Wnek et al., 2013). Indeed it is possible that the small amounts of nano-rods that are observed may arise from the incorporation of subunits from which the His-tag has been proteolytically removed. In any case, the levels of nano-rods obtained were too low to permit further detailed characterization.

**FIGURE 6:**
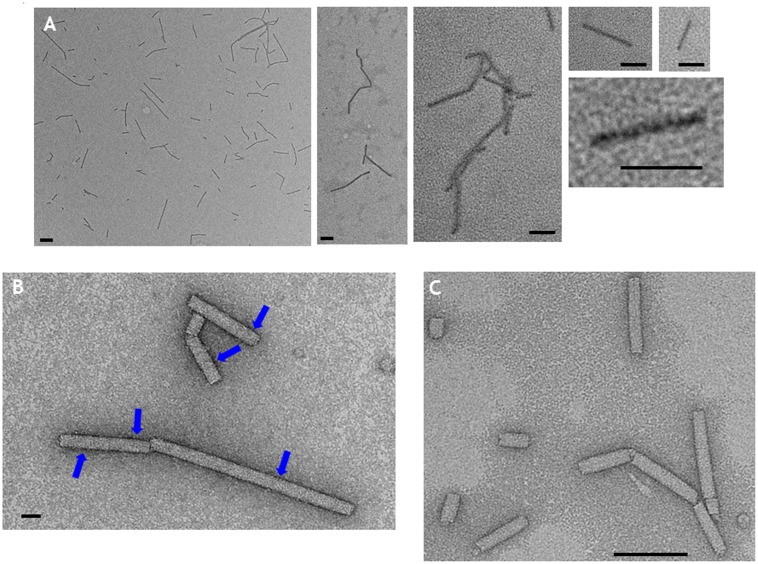
Transmission electron microscopy of CoPt-bound CP9 nano-rods. **(A)** CP9 nano-rods visualized without negative staining. **(B)** CP9 nano-rods visualized with negative staining. **(C)** WT nano-rods negatively stained. Blue arrows indicate regions of the CP9 nano-rods that appear intense indicative of CoPt deposition. Scale bar; **(A)** 100 nm, **(B)** 20 nm, **(C)** 100 nm.

By contrast, extracts of leaves infiltrated with pEAQ-*HT*-TMV-CP(CP9)/OAS appeared to contain sufficient material to allow further purification. After centrifugation of PEG-precipitated extracts from equal masses of leaves infiltrated with pEAQ-*HT*-TMV-CP/OAS or pEAQ-*HT*-TMV-CP(CP9)/OAS through CsCl gradients, the yield of the CP9 nano-rod was estimated to be approximately one third that of the WT nano-rod as judged by Instant Blue staining of coat protein resolved in NuPAGE gels (**Supplementary Figure S2**). CP9 nano-rods were morphological indistinguishable from the WT nano-rods when examined by transmission electron microscopy after negative staining with uranyl acetate (**Supplementary Figure S3**). Following CoPt deposition, CP9 nano-rods could be visualized without negative staining (**Figure 6A**). In this case, the nano-rods had a dark rod-like appearance but with a hazy outline. Intense darker regions on the CP9 nano-rods can also be seen without staining, especially at higher magnifications (**Figure 6A**). By contrast, nano-rods produced from WT coat protein could not be visualized by transmission electron microscopy unless the samples were subjected to negative staining (**Figure 6C**), even after being subjected to the CoPt deposition reaction. Negative staining of the CP9 nano-rods following CoPt deposition revealed the characteristic nano-rod structure (**Figure 6B**). The build-up of density, on the surface of the CP9 nano-rods is also evident (**Figure 6B**; blue arrows) which correlates with the darker regions observed on the unstained CP9 nano-rods (**Figure 6A**). These darker regions were more evident in the samples which underwent a longer deposition reaction (69 h) compared with those which were reacted for a shorter time, (22 h).

In this paper we have demonstrated that it is possible to synthesize defined length nano-rods by transiently expressing the TMV coat protein and an RNA containing the OAS element. These designer nano-rods have the general morphology of native TMV rods whose length can be controlled. In addition, they will lack the ability to cause an infection since the genomic RNA is not present. The ability to control their length, makes these synthetic nano-rods ideal for many emerging nanobiotechnological applications. In recent times, the concept of using plants as biofactories for the development and production of suitable pharmaceutical proteins has been gaining strength and is now very much a commercial reality (Lomonossoff and D’Aoust, 2016). With such facilities, any problematical low yielding but modified nano-rods can easily be overcome, simply by increasing the efficiency of production. As we have demonstrated by genetic modification to the coat protein outer surface, it is possible to direct the synthesis of “mutant” nano-rods whose surface properties will allow for them to bind metals or functional proteins. Though we have demonstrated the production of linear rods, further applications are possible. For example, the *in vitro* formation of nanoboomerangs and tetrapods, formed upon RNAs with more than one OAS element has been demonstrated (Eber et al., 2015) and we anticipate that similar structures could be synthesized transiently in plants following the procedures described in this paper. In addition it is possible to incorporate modified versions of the TMV coat protein into rods of predetermined size without the need either for preserving viral infectivity or having to undertake *in vitro* reassembly experiments. The ability to metallise rods of different sizes, as demonstrated in the case of CoPt, increases the scope for the deployment of TMV-based structures in nanoelectronics.

## Author Contributions

Conceived and designed the experiments: KS and GL. Performed the experiments: KS. Analyzed the data: KS and GL. Wrote the paper: KS and GL.

## Conflict of Interest Statement

GL declares that he is a named inventor on granted patent WO 29087391 A1 which describes the pEAQ vector system used for the work described in this manuscript. The other author declares that the research was conducted in the absence of any commercial or financial relationships that could be construed as a potential conflict of interest.

## Supplementary Material

**FIGURE S1 |** **(A)** Nucelotide sequence of the synthetic DNA that was cloned into pEAQ-*HT* to yield pEAQ-*HT*-TMV-CP/OAS. **(B)** Nucleotide sequence of the synthetic DNA that was cloned into pEAQ-*HT* to yield pEAQ-*HT*-TMV-CP(CP9)/OAS. The relevant restriction sites used in this cloning and subsequent DNA manipulations are highlighted in bold text.

**FIGURE S2 |** Separation of TMV, WT, and CP9 nano-rods by centrifugation in 10–50% step sucrose gradient. Samples were spun at 145,000 × *g* for 1 h at 5°C and fractionated into 300 μL fractions and 10 μL of each fraction was resolved on 12% NuPAGE gels, with the top fraction of the gradient on the left of each gel. TMV CP was visualized by Instant Blue staining. **(A)** TMV; **(B)** CP9 nano-rods; and **(C)** WT nano-rods.

**FIGURE S3 |** Visualization of UA stained nano-rod structures. **(A)** TMV; **(B)** CP9 nano-rods; and **(C)** WT nano-rods by transmission electron microscopy.

**FIGURE S4 |** Un-edited photograph of CsCl gradients. A cut down portion, tube 3 was used in **Figure 2A**. Un-edited photographs of two original gels used to make **Figure 2B**. The original positions of lanes 1–4 and M are indicated.

**FIGURE S5 |** Un-edited photograph of the ethidium bromide stained gel used in **Figure 4A**. Used lanes, displayed in **Figure 4** are indicated. Portions between them were removed for the final figure.

**FIGURE S6 |(A)** Original photograph of ethidium bromide-stained gel showing the RNA size markers (Invitrogen, catalog number 15623-200) run (Lane M) with samples that were subsequently transferred to a nylon membrane to create **Figure 4B**. **(B)** Original photograph of the northern blot, lanes 1–5, displayed in **Figure 4B**. **(C)** Denaturing agarose gel of Ambion Millenium RNA Markers, catalog number AM 7150 (Lane 1) and 1 μg TMV RNA (lane 2) stained with ethidium bromide (right) and subsequently subjected to northern blot analysis using the OAS-specific probe (left). Only the TMV RNA can be detected with the probe.

**FIGURE S7 |(A)** Original photograph of the anti TMV western blot used in **Figure 5A**. **(B)** Instant blue NuPAGE stained gel. Used lanes for **Figure 5** are indicated.
