# Supplementary figures and images for: In Planta Synthesis of Designer-Length Tobacco Mosaic Virus-Based Nano-Rods That Can Be Used to Fabricate Nano-Wires
Source: Front Plant Sci. 2017 Aug 18;8:1335. doi: 10.3389/fpls.2017.01335 (PMC5572394; doi:10.3389/fpls.2017.01335)

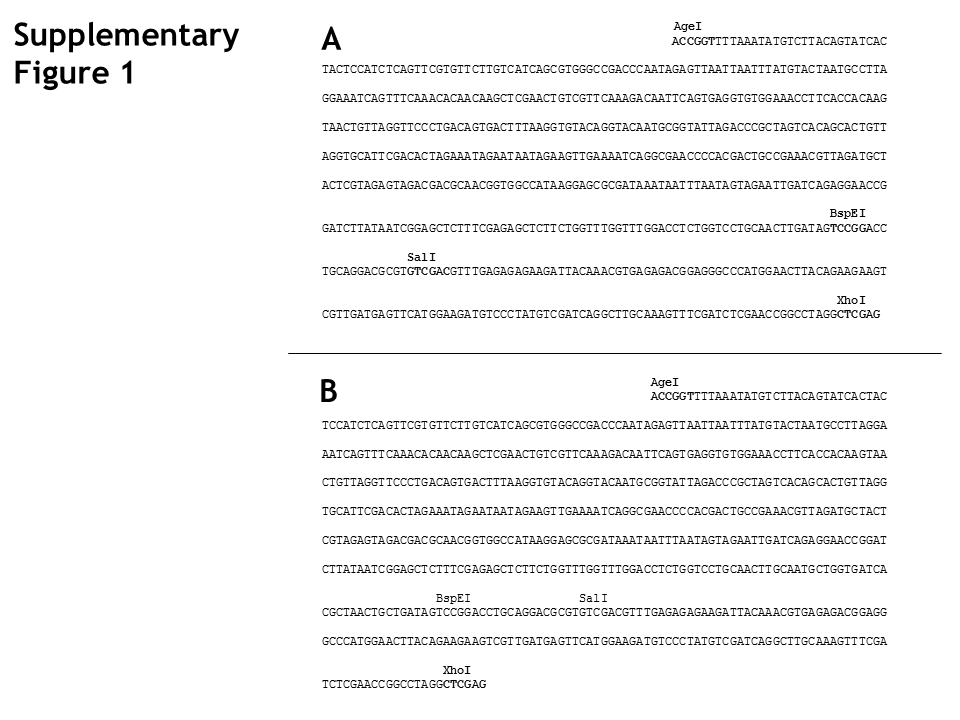

Supplement: Supplementary file 1 [file Image_1.TIF]

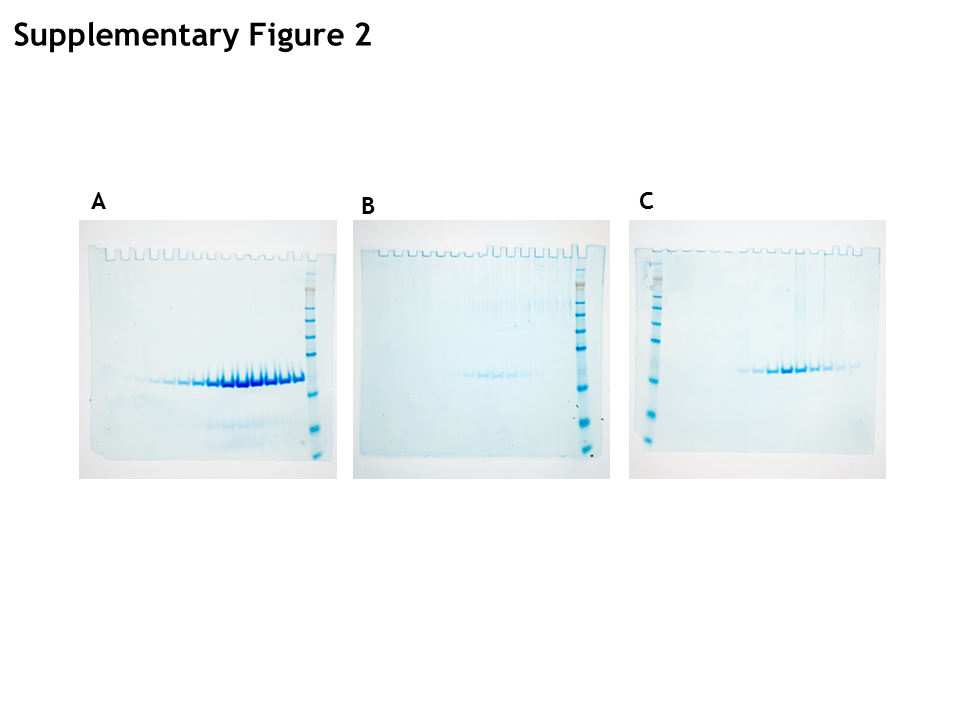

Supplement: Supplementary file 2 [file Image_2.TIF]

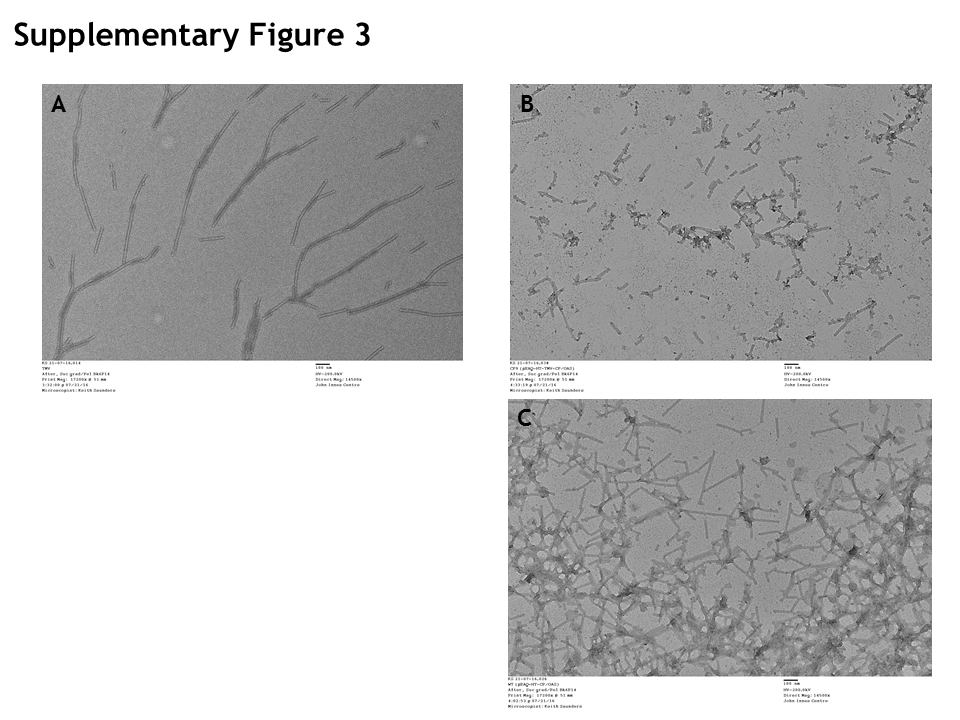

Supplement: Supplementary file 3 [file Image_3.TIF]

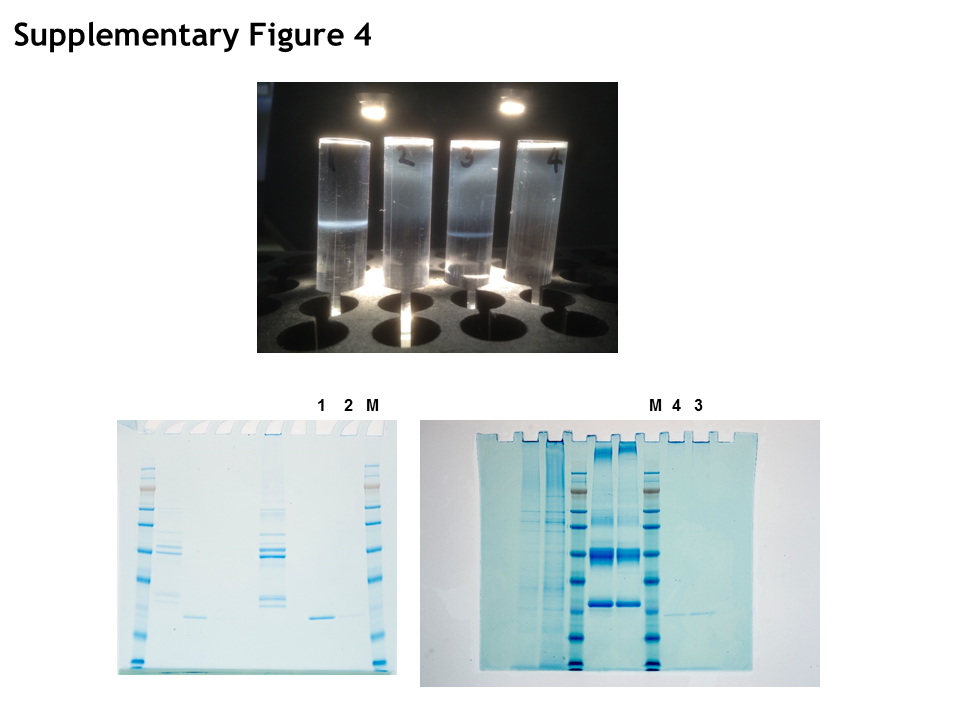

Supplement: Supplementary file 4 [file Image_4.TIF]

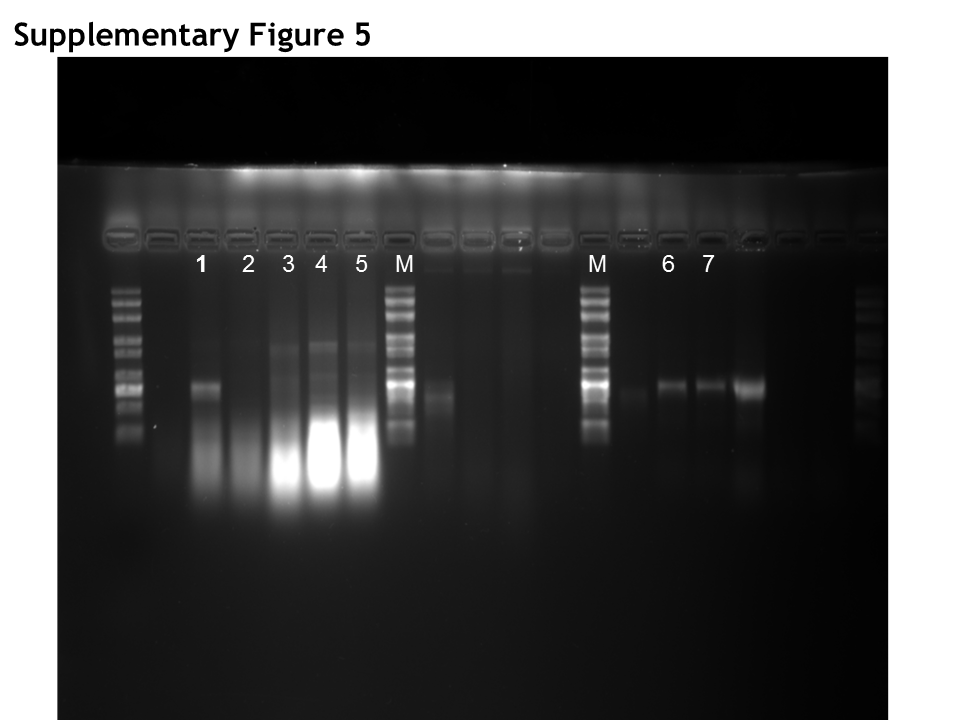

Supplement: Supplementary file 5 [file Image_5.TIF]

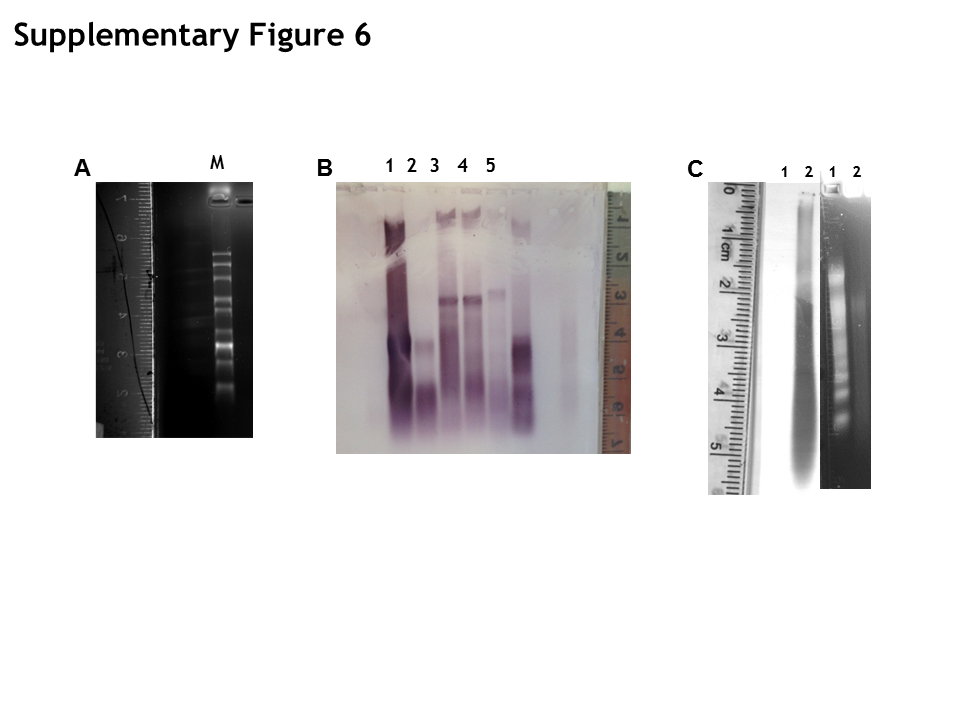

Supplement: Supplementary file 6 [file Image_6.TIF]

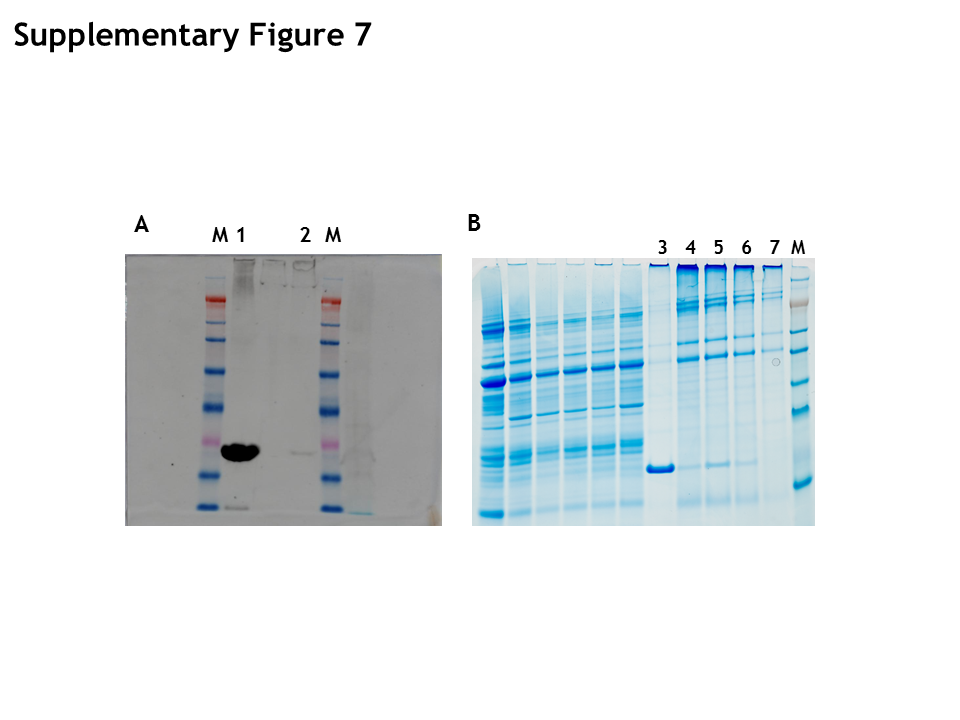

Supplement: Supplementary file 7 [file Image_7.TIF]
