# Supplementary material for: In Planta Synthesis of Designer-Length Tobacco Mosaic Virus-Based Nano-Rods That Can Be Used to Fabricate Nano-Wires
Source: Front Plant Sci. 2017 Aug 18;8:1335. doi: 10.3389/fpls.2017.01335 (PMC5572394; doi:10.3389/fpls.2017.01335)
Supplement: Supplementary file 8 [file Table_1.DOCX]

**Supplementary Table 1**

**Measurements of nano-rod length (nm)**

| **A** |  | **B** |  | **D** |  | **C** |
| --- | --- | --- | --- | --- | --- | --- |
| **Measurements in nm** | | **Measurements in nm** | | **Measurements in nm** | | **Measurements in nm** |
| **83. Entries Total** | | **62. Entries Total** | | **147. Entries Total** | | **208. Entries Total** |
| **Mean = 76.74337 nm** | | **Mean = 164.4097 nm** | | **Mean = 208.0796 nm** | | **Mean = 152.0918 nm** |
| **Dev(rms)= 43.03682 nm** | | **Dev(rms)= 117.4866 nm** | | **Dev(rms)= 89.69195 nm** | | **Dev(rms)= 47.74556 nm** |
| **length (nm)** |  | **length** |  | **Length** |  | **169** |
| **96.3** |  | **87.2** |  | **184** |  | **116** |
| **110** |  | **85.5** |  | **148** |  | **96.8** |
| **52.4** |  | **157** |  | **118** |  | **158** |
| **58.2** |  | **152** |  | **205** |  | **153** |
| **86.6** |  | **245** |  | **275** |  | **207** |
| **117** |  | **150** |  | **252** |  | **121** |
| **137** |  | **106** |  | **434** |  | **114** |
| **90.1** |  | **140** |  | **267** |  | **100** |
| **48.4** |  | **92.7** |  | **226** |  | **182** |
| **55.4** |  | **100** |  | **234** |  | **81.6** |
| **134** |  | **150** |  | **308** |  | **159** |
| **117** |  | **60.5** |  | **226** |  | **251** |
| **133** |  | **107** |  | **358** |  | **132** |
| **55.2** |  | **87** |  | **197** |  | **131** |
| **38.7** |  | **79.2** |  | **130** |  | **143** |
| **52.3** |  | **213** |  | **218** |  | **156** |
| **78.5** |  | **103** |  | **247** |  | **150** |
| **76** |  | **64** |  | **413** |  | **215** |
| **53.4** |  | **53.8** |  | **555** |  | **153** |
| **74.9** |  | **100** |  | **106** |  | **105** |
| **95.6** |  | **83.5** |  | **160** |  | **301** |
| **158** |  | **144** |  | **77.8** |  | **149** |
| **26.8** |  | **133** |  | **205** |  | **138** |
| **29.7** |  | **77.4** |  | **135** |  | **125** |
| **80.6** |  | **207** |  | **112** |  | **127** |
| **80.8** |  | **92.3** |  | **204** |  | **190** |
| **47.5** |  | **355** |  | **283** |  | **179** |
| **173** |  | **105** |  | **170** |  | **112** |
| **109** |  | **567** |  | **289** |  | **173** |
| **45** |  | **250** |  | **158** |  | **167** |
| **52.3** |  | **382** |  | **145** |  | **141** |
| **88** |  | **85.2** |  | **197** |  | **281** |
| **90.1** |  | **274** |  | **230** |  | **132** |
| **43.9** |  | **104** |  | **176** |  | **123** |
| **39.7** |  | **312** |  | **300** |  | **133** |
| **30.2** |  | **430** |  | **271** |  | **152** |
| **139** |  | **58.7** |  | **105** |  | **168** |
| **33.3** |  | **105** |  | **292** |  | **119** |
| **199** |  | **146** |  | **144** |  | **112** |
| **79.8** |  | **607** |  | **225** |  | **139** |
| **48.2** |  | **43.8** |  | **168** |  | **115** |
| **28** |  | **46.4** |  | **177** |  | **297** |
| **55** |  | **148** |  | **217** |  | **138** |
| **40.1** |  | **104** |  | **307** |  | **142** |
| **96.9** |  | **56.1** |  | **182** |  | **130** |
| **63.9** |  | **192** |  | **103** |  | **137** |
| **55.3** |  | **98.7** |  | **225** |  | **209** |
| **49.8** |  | **110** |  | **315** |  | **165** |
| **38.6** |  | **185** |  | **141** |  | **92.4** |
| **31.3** |  | **200** |  | **213** |  | **165** |
| **15.9** |  | **108** |  | **138** |  | **161** |
| **15** |  | **115** |  | **271** |  | **155** |
| **62.2** |  | **150** |  | **162** |  | **137** |
| **66.7** |  | **175** |  | **233** |  | **180** |
| **55.8** |  | **249** |  | **164** |  | **96.1** |
| **152** |  | **406** |  | **304** |  | **72.8** |
| **79.4** |  | **151** |  | **67.9** |  | **130** |
| **112** |  | **127** |  | **164** |  | **149** |
| **37.3** |  | **164** |  | **213** |  | **151** |
| **59.8** |  | **316** |  | **261** |  | **172** |
| **46.4** |  | **84.4** |  | **217** |  | **73.1** |
| **54.6** |  | **113** |  | **179** |  | **153** |
| **81.9** |  |  |  | **292** |  | **138** |
| **221** |  |  |  | **162** |  | **113** |
| **89.1** |  |  |  | **196** |  | **163** |
| **79.6** |  |  |  | **149** |  | **154** |
| **61.6** |  |  |  | **199** |  | **168** |
| **48** |  |  |  | **391** |  | **125** |
| **216** |  |  |  | **118** |  | **291** |
| **84.3** |  |  |  | **131** |  | **165** |
| **75.7** |  |  |  | **165** |  | **154** |
| **93.3** |  |  |  | **265** |  | **187** |
| **10.3** |  |  |  | **199** |  | **178** |
| **22.4** |  |  |  | **198** |  | **147** |
| **70.1** |  |  |  | **186** |  | **145** |
| **96.5** |  |  |  | **272** |  | **151** |
| **67.7** |  |  |  | **186** |  | **194** |
| **52.6** |  |  |  | **148** |  | **103** |
| **78.2** |  |  |  | **174** |  | **137** |
| **88.4** |  |  |  | **334** |  | **165** |
| **130** |  |  |  | **168** |  | **125** |
| **90.5** |  |  |  | **491** |  | **180** |
| **42.6** |  |  |  | **163** |  | **179** |
|  |  |  |  | **258** |  | **96.6** |
|  |  |  |  | **258** |  | **116** |
|  |  |  |  | **430** |  | **183** |
|  |  |  |  | **241** |  | **227** |
|  |  |  |  | **382** |  | **226** |
|  |  |  |  | **309** |  | **104** |
|  |  |  |  | **233** |  | **200** |
|  |  |  |  | **184** |  | **172** |
|  |  |  |  | **164** |  | **165** |
|  |  |  |  | **134** |  | **187** |
|  |  |  |  | **131** |  | **145** |
|  |  |  |  | **243** |  | **210** |
|  |  |  |  | **346** |  | **152** |
|  |  |  |  | **113** |  | **116** |
|  |  |  |  | **139** |  | **299** |
|  |  |  |  | **225** |  | **178** |
|  |  |  |  | **144** |  | **170** |
|  |  |  |  | **170** |  | **121** |
|  |  |  |  | **160** |  | **130** |
|  |  |  |  | **236** |  | **154** |
|  |  |  |  | **415** |  | **150** |
|  |  |  |  | **142** |  | **91.8** |
|  |  |  |  | **38.5** |  | **249** |
|  |  |  |  | **239** |  | **161** |
|  |  |  |  | **330** |  | **61.9** |
|  |  |  |  | **142** |  | **161** |
|  |  |  |  | **163** |  | **161** |
|  |  |  |  | **76.5** |  | **230** |
|  |  |  |  | **205** |  | **268** |
|  |  |  |  | **183** |  | **91.7** |
|  |  |  |  | **81.5** |  | **91.7** |
|  |  |  |  | **169** |  | **90.5** |
|  |  |  |  | **364** |  | **129** |
|  |  |  |  | **122** |  | **189** |
|  |  |  |  | **50.8** |  | **79.7** |
|  |  |  |  | **244** |  | **80.5** |
|  |  |  |  | **140** |  | **95.6** |
|  |  |  |  | **205** |  | **114** |
|  |  |  |  | **253** |  | **158** |
|  |  |  |  | **174** |  | **177** |
|  |  |  |  | **204** |  | **102** |
|  |  |  |  | **161** |  | **95.7** |
|  |  |  |  | **403** |  | **76.2** |
|  |  |  |  | **155** |  | **119** |
|  |  |  |  | **270** |  | **122** |
|  |  |  |  | **153** |  | **218** |
|  |  |  |  | **258** |  | **144** |
|  |  |  |  | **217** |  | **117** |
|  |  |  |  | **99.4** |  | **126** |
|  |  |  |  | **332** |  | **76.7** |
|  |  |  |  | **142** |  | **176** |
|  |  |  |  | **124** |  | **145** |
|  |  |  |  | **320** |  | **150** |
|  |  |  |  | **91.9** |  | **136** |
|  |  |  |  | **201** |  | **267** |
|  |  |  |  | **97.8** |  | **149** |
|  |  |  |  | **172** |  | **152** |
|  |  |  |  | **168** |  | **198** |
|  |  |  |  | **81.8** |  | **168** |
|  |  |  |  | **64.2** |  | **102** |
|  |  |  |  | **76.6** |  | **154** |
|  |  |  |  | **157** |  | **74.7** |
|  |  |  |  | **207** |  | **130** |
|  |  |  |  | **206** |  | **113** |
|  |  |  |  |  |  | **116** |
|  |  |  |  |  |  | **168** |
|  |  |  |  |  |  | **147** |
|  |  |  |  |  |  | **113** |
|  |  |  |  |  |  | **136** |
|  |  |  |  |  |  | **198** |
|  |  |  |  |  |  | **157** |
|  |  |  |  |  |  | **169** |
|  |  |  |  |  |  | **117** |
|  |  |  |  |  |  | **136** |
|  |  |  |  |  |  | **194** |
|  |  |  |  |  |  | **138** |
|  |  |  |  |  |  | **194** |
|  |  |  |  |  |  | **179** |
|  |  |  |  |  |  | **146** |
|  |  |  |  |  |  | **143** |
|  |  |  |  |  |  | **159** |
|  |  |  |  |  |  | **119** |
|  |  |  |  |  |  | **123** |
|  |  |  |  |  |  | **145** |
|  |  |  |  |  |  | **126** |
|  |  |  |  |  |  | **186** |
|  |  |  |  |  |  | **110** |
|  |  |  |  |  |  | **152** |
|  |  |  |  |  |  | **202** |
|  |  |  |  |  |  | **73** |
|  |  |  |  |  |  | **277** |
|  |  |  |  |  |  | **150** |
|  |  |  |  |  |  | **111** |
|  |  |  |  |  |  | **238** |
|  |  |  |  |  |  | **218** |
|  |  |  |  |  |  | **235** |
|  |  |  |  |  |  | **243** |
|  |  |  |  |  |  | **123** |
|  |  |  |  |  |  | **136** |
|  |  |  |  |  |  | **174** |
|  |  |  |  |  |  | **106** |
|  |  |  |  |  |  | **109** |
|  |  |  |  |  |  | **77.2** |
|  |  |  |  |  |  | **97.1** |
|  |  |  |  |  |  | **113** |
|  |  |  |  |  |  | **222** |
|  |  |  |  |  |  | **193** |
|  |  |  |  |  |  | **143** |
|  |  |  |  |  |  | **134** |
|  |  |  |  |  |  | **73.9** |
|  |  |  |  |  |  | **133** |
|  |  |  |  |  |  | **154** |
|  |  |  |  |  |  | **192** |
|  |  |  |  |  |  | **193** |
|  |  |  |  |  |  | **167** |
|  |  |  |  |  |  | **278** |
|  |  |  |  |  |  | **223** |
|  |  |  |  |  |  | **214** |
|  |  |  |  |  |  | **152** |
|  |  |  |  |  |  | **126** |
|  |  |  |  |  |  | **110** |
|  |  |  |  |  |  | **91.8** |
|  |  |  |  |  |  | **147** |
|  |  |  |  |  |  | **105** |
